# Combined assessment of ΔPCT and ΔCRP could increase the ability to differentiate candidemia from bacteremia

**DOI:** 10.1186/s13054-019-2557-8

**Published:** 2019-08-05

**Authors:** Qin Wu, Hao Yang, Yan Kang

**Affiliations:** Department of Critical Care Medicine, West China Hospital, Sichuan University, Chengdu, China

Dear editor,

We read with great interest the paper from Cortegiani et al. and agreed with their conclusion that procalcitonin (PCT) should not be used as a standalone tool for differential diagnosis between candidemia and bacteremia [1]. In theory, PCT concentration changes more rapidly than C-reactive protein (CRP) in response to bacterial infection and appropriate antibiotic therapy appears to be correlated with a rapid decrease in PCT level [2, 3]. It might thus be helpful to differentiate candidemia from bacteremia based on changes in both biomarkers over time.

To test this hypothesis, we retrospectively enrolled a subset of patients who were discharged from our department during the period from Jan. 1, 2016, to Dec. 31, 2018; these patients had suspected bloodstream infection at the time of intensive care unit (ICU) admission, and blood samples had been drawn for culture in accordance with our hospital’s standard protocol, as well as PCT and CRP have been measured at admission and 2 days after admission. Only patients > 18 years of age who had been administered both appropriate antifungal and antimicrobial empirical treatments with enough data for further analysis were included in the study. For culture-positive patients, antifungal and antimicrobial treatment was classified as being appropriate if the initially prescribed antibiotic regimen was active against the identified pathogen based on in vitro susceptibility testing. For culture-negative patients, initial antibiotic therapy was defined as appropriate if it complied with the recommendations of the current local guidelines for suspected bloodstream infection in patients admitted to an ICU. Bloodstream infectious episodes were defined as blood cultures that were positive according to judgment of two independent intensivists. We defined ΔPCT as the difference between PCT level on day 2 and PCT level at admission; ΔCRP was defined as the difference between CRP level on day 2 and CRP level at admission; and ΔPCT-ΔCRP was defined as ΔPCT minus ΔCRP.

A total of 190 patients were included during the study period; six had confirmed bacteremia and eight had confirmed candidemia. Baseline characteristics for all patients are shown in Additional file 1; notably, the patient groups were comparable in the assessed characteristics. Culture-positive patients had a longer length of stay in the ICU.

Figure 1 shows PCT levels, CRP levels, ΔPCT, ΔCRP, and ΔPCT-ΔCRP between bacteremia, candidemia, and culture-negative patients. The area under the curve (AUC) for ΔPCT-ΔCRP was 0.813 (95% confidence interval (CI) 0.541–1.000, *p* = 0.043); this was greater than the AUCs of PCT (AUC 0.573, 95% CI 0.240–0.906, *p* = 0.651), CRP (AUC 0.604, 95% CI 0.287–0.921, *p* = 0.519), ΔPCT (AUC 0.563, 95% CI 0.195–0.930, *p* = 0.699), and ΔCRP (AUC 0.604, 95% CI 0.292–0.916, *p* = 0.519). Patients with ΔPCT-ΔCRP > 50.49 have a sensitivity of 61.53% and a specificity of 60% for predicting candidemia. To the best of our knowledge, this is the first investigation of the combined predictive abilities of changes in PCT and changes in other markers. Our data suggest that combined assessment of ΔPCT and ΔCRP could increase the predictive value of these parameters and enhance differentiation of candidemia from bacteremia among patients who received both appropriate antifungal and antimicrobial empirical treatment. Further prospective studies are needed to confirm our findings in a larger population and in patients without both appropriate antifungal and antimicrobial empirical treatment.

**Fig. 1:**
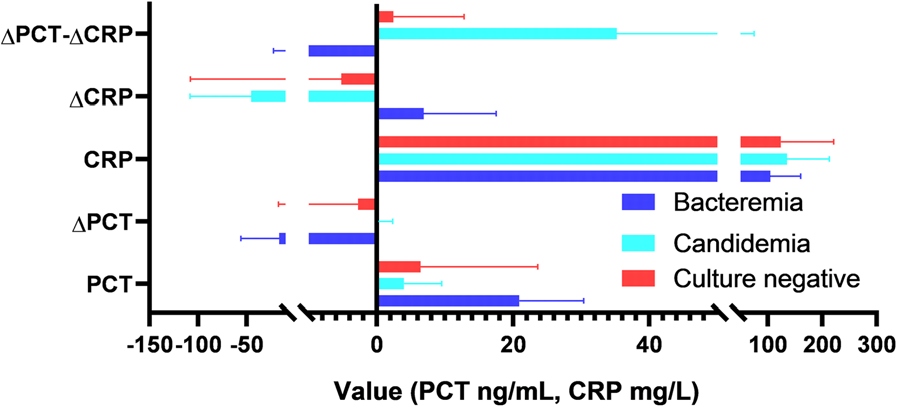
Plot of procalcitonin (PCT) at admission, C-reactive protein (CRP) at admission, ΔPCT (defined as PCT level on day 2 minus PCT level at admission), ΔCRP (defined as CRP level on day 2 minus CRP level at admission), and ΔPCT-ΔCRP (defined as ΔPCT minus ΔCRP) in candidemia, bacteremia, and culture-negative patients

## Additional file

Additional file 1: **Table S1.** Demographics, clinical and outcome data of patient cohort. (DOCX 27 kb)

## Data Availability

The datasets generated and analyzed in this article are not publicly available due to health privacy concerns. However, they are available from the corresponding author and will be obtainable by the public when the database construction is complete.

## Abbreviations

AUC: Area under the curve
CI: Confidence interval
CRP: C-reactive protein
ICU: Intensive care unit
PCT: Procalcitonin

## References

[1] Cortegiani A, Misseri G, Ippolito M, Bassetti M, Giarratano A, Martin-Loeches I, Einav S (2019). Procalcitonin levels in candidemia versus bacteremia: a systematic review. Crit Care.

[2] Massaro KS, Costa SF, Leone C, Chamone DA (2007). Procalcitonin (PCT) and C-reactive protein (CRP) as severe systemic infection markers in febrile neutropenic adults. BMC Infect Dis.

[3] Viallon A, Guyomarc'h P, Guyomarc'h S, Tardy B, Robert F, Marjollet O, Caricajo A, Lambert C, Zeni F, Bertrand JC (2005). Decrease in serum procalcitonin levels over time during treatment of acute bacterial meningitis. Crit Care.

