# Supplementary material for: Combined assessment of ΔPCT and ΔCRP could increase the ability to differentiate candidemia from bacteremia
Source: Crit Care. 2019 Aug 5;23:271. doi: 10.1186/s13054-019-2557-8 (PMC6681475; doi:10.1186/s13054-019-2557-8)
Supplement: Supplementary file 1 — Table S1. Demographics, clinical and outcome data of patient cohort. (DOCX 27 kb) [file 13054_2019_2557_MOESM1_ESM.docx]

**Table S1 Demographics, Clinical and Outcome data of patient cohort**

| **Parameters** | **All**  **(n=190)** | **Culture negative**  **(n=176)** | **Culture positive** | | | **P*^a^*** | **P*^b^*** |
| --- | --- | --- | --- | --- | --- | --- | --- |
|  |  |  | **All (14)** | **Bacteremia (n=6)** | **Candidemia (n=8)** |  |  |
| Sociodemographic |  |  |  |  |  |  |  |
| Age, mean±SD, year | 56.29±16.79 | 56.17±16.86 | 57.86±16.38 | 59.0±20.07 | 57.0±14.44 | 0.719 | 0.831 |
| Male, n (%) | 102 (53.68) | 95 (53.98) | 7 (50.0) | 2 (33.33) | 5 (62.5) | 0.993 | 0.589 |
| APACHE Ⅱ Score, mean±SD | 19.87±6.82 | 20.04±6.82 | 17.69±6.8 | 14.20±8.41 | 19.88±4.97 | 0.251 | 0.150 |
| Outcome |  |  |  |  |  |  |  |
| Hospital LOS, mean±SD, day | 23.86±13.69 | 23.63±13.59 | 26.79±15.15 | 34.83±18.04 | 20.75±9.84 | 0.408 | 0.084 |
| ICU LOS, mean±SD, day | 14.16±10.25 | 13.73±9.68 | 19.52±15.27 | 25.79±20.93 | 14.82±7.83 | 0.042 | 0.194 |
| In-hospital mortality, n(%) | 80 (42.11) | 73 (41.48) | 7 (50.0) | 3 (50.0) | 4 (50.0) | 0.734 | 1.000 |
| Inflammation marker* |  |  |  |  |  |  |  |
| PCT, mean±SD, ng/mL | 7.52±19.22 | 7.82±19.9 | 3.67±4.85 | 5.75±6.81 | 2.11±2.04 | 0.438 | 0.174 |
| CRP, mean±SD, mg/L | 109.62±100.81 | 110.67±102.33 | 96.33±81.05 | 57.23±58.8 | 125.66±86.24 | 0.610 | 0.121 |
| Complete blood count* |  |  |  |  |  |  |  |
| RBC, mean±SD,×10^9^/L | 3.4±0.81 | 3.42±0.81 | 3.12±0.78 | 3.75±0.75 | 2.65±0.37 | 0.191 | 0.003 |
| HGB, mean (SD), g/L | 100.0±23.73 | 100.62±23.67 | 92.21±23.92 | 109.83±26.21 | 79.0±10.32 | 0.203 | 0.010 |
| HCT, mean (SD), L/L | 0.31±0.07 | 0.31±0.07 | 0.29±0.07 | 0.33±0.08 | 0.25±0.03 | 0.253 | 0.018 |
| PLT, mean (SD),×10^9^/L | 146.64±92.22 | 143.19±91.42 | 189.93±94.62 | 130.0±73.8 | 234.88±85.74 | 0.068 | 0.031 |
| WBC, mean (SD),×10^9^/L | 11.45±6.3 | 11.4±6.09 | 12.07±8.73 | 8.55±2.88 | 14.72±10.81 | 0.702 | 0.202 |
| Coagulation test* |  |  |  |  |  |  |  |
| INR, mean (SD) | 1.30±0.39 | 1.29±0.37 | 1.47±0.56 | 1.36±0.43 | 1.56±0.66 | 0.081 | 0.536 |
| PT, mean (SD), s | 15.24±4.42 | 15.09±4.21 | 17.23±6.41 | 15.93±4.94 | 18.2±7.51 | 0.081 | 0.534 |
| APTT, mean (SD), s | 41.51±19.43 | 41.27±19.76 | 44.51±14.89 | 42.77±18.42 | 45.83±12.84 | 0.455 | 0.720 |
| Fib, mean (SD), g/L | 3.57±1.81 | 3.58±1.84 | 3.39±1.4 | 2.85±1.6 | 3.80±1.17 | 0.715 | 0.220 |
| Arterial Blood Gas Test* |  |  |  |  |  |  |  |
| pH, mean (SD) | 7.10±0.69 | 7.10±0.69 | 7.14±0.73 | 7.20±0.59 | 7.09±0.86 | 0.848 | 0.783 |
| PaO_2_, mean (SD), mmHg | 102.19±46.26 | 102.1±44.12 | 103.16±69.39 | 87.38±16.66 | 115.00±91.49 | 0.935 | 0.483 |
| PaCO_2_, mean (SD), mmHg | 39.27±8.78 | 39.06±8.68 | 41.75±9.89 | 37.05±3.6 | 45.28±11.81 | 0.341 | 0.128 |
| BE, mean (SD), mmol/L | 3.38±3.78 | 3.21±3.62 | 4.52±4.77 | 1.92±1.8 | 6.00±5.4 | 0.401 | 0.185 |
| Lac, mean (SD), mmol/L | 2.19±1.58 | 2.21±1.6 | 2.02±1.46 | 1.46±0.54 | 2.37±1.77 | 0.671 | 0.291 |
| Biochemical analysis* |  |  |  |  |  |  |  |
| Serum sodium, mean (SD), mmol/L | 140.07±7.21 | 140.14±7.31 | 139.19±5.98 | 139.55±3.66 | 138.91±7.53 | 0.634 | 0.853 |
| Serum potassium, mean (SD), mmol/L | 3.95±0.6 | 3.95±0.61 | 3.98±0.57 | 3.71±0.5 | 4.19±0.56 | 0.839 | 0.121 |
| Serum chloride, mean (SD), mmol/L | 108.0±7.88 | 108.35±7.71 | 103.58±8.9 | 107.15±8.76 | 100.9±8.55 | 0.029 | 0.210 |
| TB, mean (SD), mmol/L | 23.83±32.5 | 23.41±32.95 | 29.09±26.69 | 36.20±20.63 | 23.76±30.72 | 0.530 | 0.383 |
| TP, mean (SD), mmol/L | 51.92±9.01 | 51.82±9.14 | 53.21±7.35 | 50.15±9.17 | 55.5±5.12 | 0.581 | 0.188 |
| Creatinine, mean (SD), mmol/L | 108.46±99.73 | 111.73±102.57 | 67.29±31.54 | 72.67±30.24 | 63.25±33.93 | 0.109 | 0.595 |
| Cys-c, mean (SD), mmol/L | 1.23±0.77 | 1.25±0.79 | 1.08±0.52 | 0.87±0.23 | 1.23±0.64 | 0.426 | 0.217 |
| GGT, mean (SD), mmol/L | 57.23±64.95 | 56.01±65.78 | 72.5±52.92 | 40.17±21.15 | 96.75±57.55 | 0.287 | 0.042 |
| TG, mean (SD), mmol/L | 1.69±1.69 | 1.68±1.73 | 1.74±1.17 | 1.53±0.87 | 1.9±1.39 | 0.905 | 0.574 |

*Measured when drawing blood for culture

RBC: Red blood cell, HGB: Hemoglobin, HCT: Hematocrit, PLT: Platelets, WBC: white blood cell; INR: International normalized ratio, PT: prothrombin time, APTT: Activated partial thromboplastin time, Fib: fibrinogen, TT: Thrombin time, PaO_2_: Arterial oxygen partial pressure, PaCO_2_: Arterial carbon dioxide partial pressure, BE: Base excess, Lac: lactate, TB: Total bilirubin, TP: Total protein, Cys-c: Cystatin C, GGT: Gamma-Glutamyl Transferase,

*^a^*:compared between culture negative and all culture positive patients

*^b^*:compared between candidemia and bacteremia
